# COPT2, a plasma membrane located copper transporter, is involved in the uptake of Au in *Arabidopsis*

**DOI:** 10.1038/s41598-017-11896-5

**Published:** 2017-09-12

**Authors:** Manish Tiwari, Perumal Venkatachalam, Lola Penarrubia, Shivendra V. Sahi

**Affiliations:** 1 Department of Biology, Western Kentucky University, 1906 College Heights, Bowling Green, 42101-1080 Kentucky USA; 2 Department of Biotechnology, Periyar University, Salem, 636 011 Tamil Nadu India; 3 Departament de Bioquímicai Biologia Molecular Facultat de Biologia Universitat de València Ave. Doctor Moliner, 50 E-46100, Burjassot, Valencia Spain; 4 Department of Plant Systems Biology, VIB, Ghent University, Gent, 9000 Belgium

## Abstract

The mechanism of gold nanoparticle formation and genes involved in such processes, especially Au transport in plants are not understood. Previous reports pointed to the probable role of COPT2 in Au transport based on the transcript accumulation of *COPT2* under Au exposure. Here, we provide evidence revealing the additional role of COPT2 for Au mobilization in yeast and *Arabidopsis*. The *COPT2* transcripts significantly accumulated in the root of *Arabidopsis* under Au exposure. The expression of *COPT2* restores Cu uptake ability in *ctr1Δctr3Δ* mutants and leads to Au sensitivity in yeast, which is comparable to Cu in growth kinetics experiments. The metal measurement data showed that the Au level was increased in *COPT2*, expressing yeast cells compared to vector transformed control. The *copt2* mutant of *Arabidopsis* displayed a similar growth pattern to that of Col-0 under Au treatment. However, a notable phenotypic difference was noticed in three-week-old plants treated with and without Au. Consistent with yeast, Au uptake was reduced in the *copt2* mutant of *Arabidopsis*. Together, these results clearly reveal the Au uptake capability of COPT2 in yeast and *Arabidopsis*. This is the first report showing the potential role of any transporter towards uptake and accumulation of Au in plants.

## Introduction

Gold (Au) and silver nanoparticles are widely used in analytical techniques, sensors, catalytic processes, and therapeutics^1^. An array of chemical and physical techniques are derived for the production of nanoparticles. Nonetheless, there are concomitant growing apprehensions concerning the risk of generating hazardous chemical byproducts that lead to environmental degradation. The use of biological organisms is an alternative method for fabricating nanoparticles, and is considered a novel way to synthesize nanoparticles^2, 3^. Therefore, the synthesis of metallic nanoparticles using plant based methods has gained momentum in recent years and became the subject of extensive research^4^. The fabrication of nanomaterials using plant-based methods is rapid, eco-friendly and easy. In addition, these nanoparticles are more stable in terms of shapes and size compared to those produced by using other organism^5^. Some plant have a natural tendency to convert the ionic Au (Au^3+^) into a reduced Au^0^ form that aggregates into different shapes and sizes of nanoparticles when entering plant cells^6, 7^. However, the knowledge of compounds and biochemical reactions that participate in such process is very limited. Several efforts have been made in recent years to address the underpinning genetic and biochemical processes for the synthesis of gold nanoparticles (AuNPs) in plants^8^. Earlier studies indicated that cellular thiols, organic acids, and similar compounds may play a prominent role in the bio-reduction of cellular ionic Au into nanoparticles^9, 10^. Studies in alfalfa revealed that various forms of Au present on the root surface^11^. However, the role of transporters involved in Au uptake is not reported yet so far in plants. Few studies in search of the uptake of ionic Au and possible mechanisms of nanoparticle formation have been conducted using the model plant *Arabidopsis thaliana* ^8–10^. The gene expression analysis indicates a modulated expression of numerous potential transporters such as *COPT2* in response to Au and implied putative role of COPT2 in the Au uptake^8–10^.

The COPT family is recognized for Cu acquisition and transport in eukaryotes including yeast, mammals, algae and plants^12–14^. The genome wide search for members showed that six homologs of the COPT family are present in the *Arabidopsis* genome^15^. The COPT1 is the first characterized member of this family for its ability to rescue Cu uptake in *ctr1Δ* yeast mutant^12^. The COPT1 is a plasma membrane localized transporter that mediates Cu influx in the root of *Arabidopsis* ^13, 16^. Similarly, COPT2 was functionally complemented *ctr1Δctr3Δ* mutant of yeast^17^. On the other hand, COPT5 lies on tonoplast and pre-vacuolar vesicles and is shown to involved in the mobilization of stored Cu by exporting outside to organelles during Cu deficiency^18, 19^. Recent characterization of COPT6 demonstrated the Cu uptake ability in the *ctr1Δctr3Δ* yeast mutant, however, it has been shown that COPT6 primarily engaged in the aerial distribution of Cu in *Arabidopsis* ^20^. In general, function of the members of the COPT family is well known for regulation of Cu transport and homeostasis in plants.

The past studies have shown that arsenate is structurally analogous to phosphate and, therefore, moves through phosphate transporters due to common structure and chemical properties^21^. Similarly, arsenite moves through Lsi1, a known transporter for silicic acid, owing to a structural resemblance in rice^22^. Our previous genome-wide expression analyses performed on short as well as long-term exposure of Au revealed mRNA accumulation of *COPT2* in the root of *Arabidopsis*, indicating the possible role of COPT2 in Au uptake^9, 10^. The Cu and Au are represented in the same group of the periodic table and share similar chemical and structural features. Thus, we raised the possibility that Au may enter through Cu transporters in *Arabidopsis*. The observations presented in this study clearly showed that COPT2, a plasma-membrane localized transporter, is responsible for the uptake of Au in the root of *Arabidopsis*.

## Results

### *COPT2* expression induced in the root by Au in a time-dependent manner

**Figure 1:**
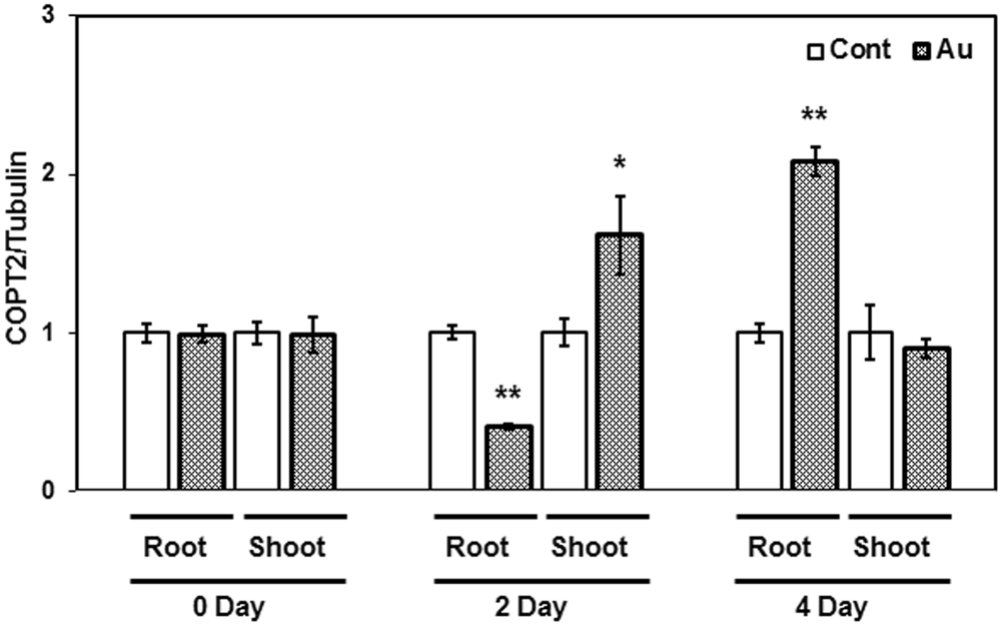
Expression profile of *COPT2* in the root and shoot of *Arabidopsis*. Three-week-old plants grown in hydroponic conditions were transferred into half strength medium (pH 5.7) with and without Au. The qRT-PCR analysis was performed at indicated time intervals. The experiment was performed at least three times, and the bars show mean of three replicate of one set. The * and ** placed on the top of the column denoting P > 0.01 and P > 0.001.

In order to determine the specific expression of *COPTs* in response to Au, gene expression kinetics of all *COPTs* were examined at different time intervals in the root and shoot of *Arabidopsis* grown in the absence and presence of Au. Three-week-old plants grown under nutrient sufficiency conditions were transferred to half-strength MS media containing KAuCl_4_ (10 ppm) for Au treatment. After immediate transfer, an initial sample designated as 0 day was collected, and thereafter, 2 and 4 day sampling was carried out. A differential expression of *COPT2* was observed at 2 and 4 days of Au exposure. The mRNA level of *COPT2* was decreased in the root while significantly increased in the shoot, corresponding to control after 2 days of Au exposure (Fig. 1). The analysis indicates that prolonged exposure of Au (4 day) specifically induced the *COPT2* transcript accumulation in the root compared to control plant (Fig. 1). A similar result was also observed in our previous study in which significant up-regulation of *COPT2* was reported in the root of *Arabidopsis* under seven days of Au exposure^9^.

The expression profile of other *COPTs* including *COPT1*, *COPT3*, *COPT4*, *COPT5*, and *COPT6* showed that the expression of these genes was also influenced by Au as indicated in 2 and 4 days of exposure (Supplementary Figure 1). These *COPTs* were mostly down-regulated in both the root and shoot tissues except *COPT3*, which was significantly induced (3.5 fold) in the shoot after 2 day of Au exposure. The expression analysis of *COPT6* showed a significant reduction of mRNA levels in the root and shoot after 2 days of exposure. However, after an initial decrease, expression reached equivalent to control in the root after 4 days of treatment (Supplementary Figure 1). In the present study, expression analysis results clearly showed that long-term Au exposure specifically enhanced the *COPT2* transcript accumulation in the root tissues of *Arabidopsis*.

### Heterologous expression of COPT2 leads to Au sensitivity in yeast

The copper transport ability of COPT2 has been extensively studied in yeast, animals, and plants. To ascertain the Au uptake potential, the *COPT2* gene was cloned from *Arabidopsis* and expressed in *ctr1Δctr3Δ* yeast double mutants under the control of the TDH3 promoter. The *ctr1Δctr3Δ* yeast strain lacks the inherent ability to survive under Cu-limiting conditions because of carrying defective high-affinity plasma membrane transporters^23^. We compared the Au transport capability of COPT2 in *ctr1Δctr3Δ* by growing it in the presence of Au with and without Cu on YPEG medium. Yeast mutants expressing functional COPT2 were grown well on YPEG media whereas empty vector transformed cells failed to survive under the same condition (Supplementary Figure 2A). However, vector containing yeast strain grew equivalent to COPT2 transformed yeast after supplementation of Cu (100 µM) in the media (Supplementary Figure 2B). There was no difference marked between the growth of the vector and COPT2-expressing yeast strain on the solid media containing either 50 or 200 µM of Au along with Cu (Supplementary Figure 2).

We also performed yeast spot assay on synthetic complete (SC) agar media. The growth of the vector and COPT2-expressing yeast cells were comparable to each other on the SC medium. The COPT2-expressing yeast exhibited sensitivity in the presence of Au with respect to the vector at higher dilutions (Supplementary Figure 3). The sensitivity in COPT2-expressing yeast appeared much more prominent at higher dilutions and concentrations of Au (300 µM) indicating that COPT2 may transport Au in yeast. The spot assay results suggest that the presence of functional COPT2 facilitates the intake of Au and leads to sensitivity in the yeast mutant.

### A significant growth inhibition observed in COPT2 expressing yeast mutant

**Figure 2:**
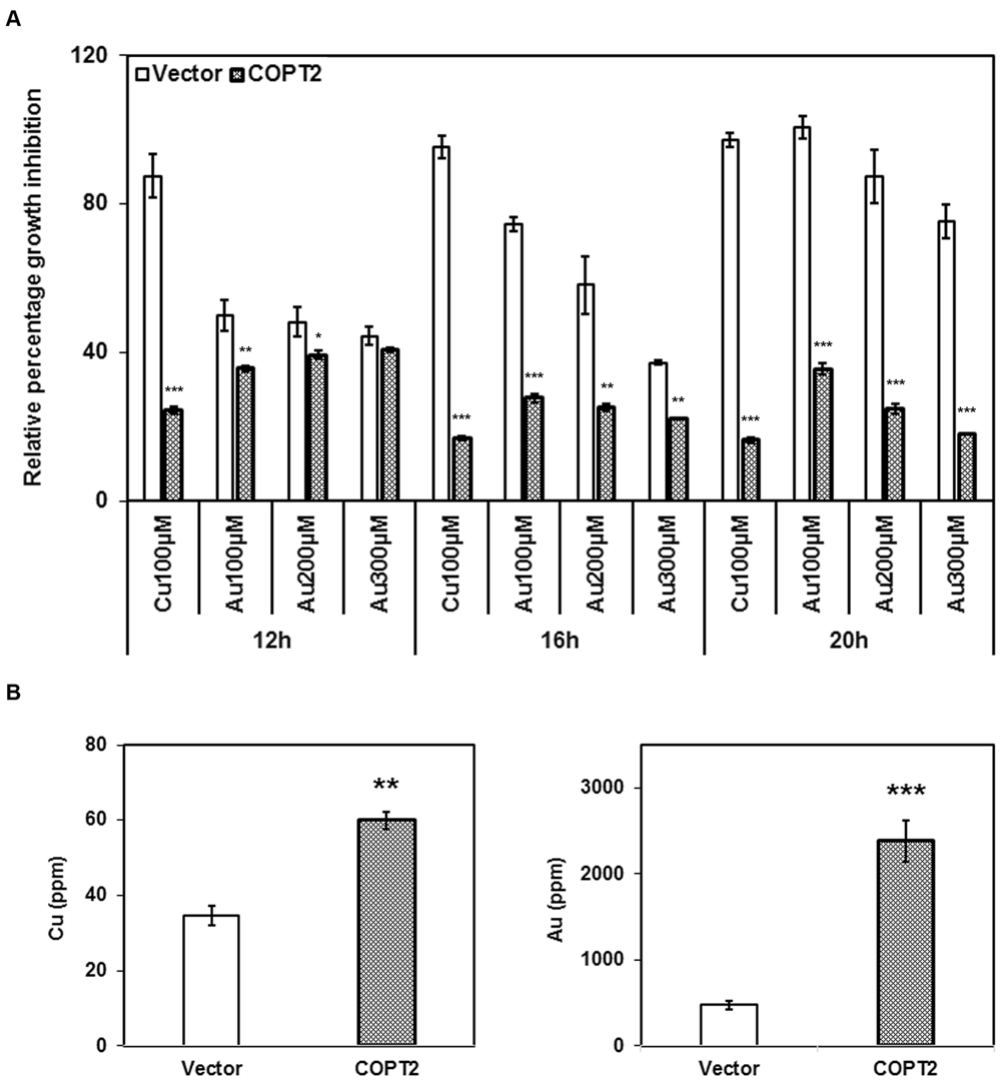
The Au-induced growth inhibition and uptake ability of COPT2 in yeast. (**A**) The precultures (O.D. 1.0) of vector and COPT2-transformed clones were inoculated in SC-URA medium with and without mentioned concentrations of Cu and Au and were monitored for growth. The data shows the relative percentage growth inhibition of three individual replicates. (**B**) Overnight grown cultures (O.D. 1.5) were fed with Au (100 µM) and allowed to grow for 4 h. Cells were harvested and washed with nanopure water many times. Elemental profiling was performed using ICP-OES. Asterisks *, ** and *** denote significant difference P > 0.01, P > 0.001 and P > 0.0001 compared to vector transformed yeast cells.

To measure toxicity caused by Au uptake, growth kinetics of the vector and *COPT2*-expressing yeast cells were investigated in liquid SC media. Cu is a well-established substrate for the members of COPT family in a variety of organisms including plants, so we then considered Cu as a positive control and included it in our kinetics study. The OD of yeast preculture was maintained at 1.0 and inoculated for secondary growth with and without Cu and Au. The final growth (OD) was recorded at 12, 16 and 20 h intervals. The percentage of relative growth data showed a growth inhibition of the COPT2-expressing yeast strain started after 12 h post inoculation compared to vector transformed cells in the presence of Cu and Au (Fig. 2A). The growth inhibition reached a maximum at 16 h and 20 h post inoculation. At these time intervals, Au (300 µM)-induced toxicity in COPT2-expressing yeast cells was equivalent to the toxicity generated by Cu (100 µM). The kinetics data also indicated that Au-induced growth inhibition was dependent on the concentrations, suggesting the role of COPT2 in the uptake of Au (Fig. 2A).

To confirm this notion, we further measured Cu and Au levels with other essential elements in yeast. The overnight grown culture of the vector and *COPT2*-expressing yeast strain (OD, 1.5) were allowed to grow further to 4 h in the presence of Au (100 µM). The elemental analysis data revealed that *COPT2-*expressing yeast cells accumulated significantly higher amount of Cu and Au content with respect to the vector transformed control (Fig. 2B). The observation also showed that the level of essential elements such as Fe and Mn also increased in contrast to Mg in *COPT2*-expressing cells compared to vector control (Supplementary Figure 4). Altogether, these results explicitly suggest that COPT2 is responsible for Au transport that is similar to Cu in the yeast mutant.

### Mutation of *COPT2* reduced Au uptake in *Arabidopsis*

**Figure 3:**
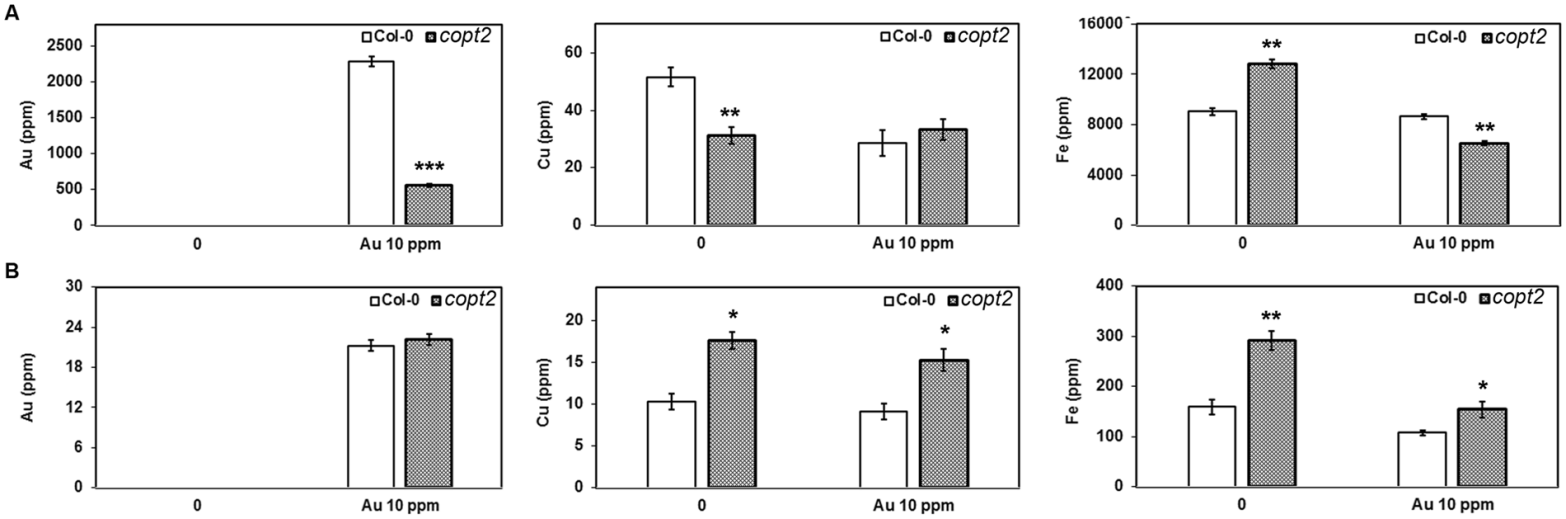
Element measurement in root and shoot of *Arabidopsis*. (**A**) Elemental profiling under control and Au (10 ppm) treated conditions through ICP in root, and (**B**) shoot. Data are a mean of three independent biological replicates and presented as ±SEM. Asterisks (*, ** and ***) represent significant difference P > 0.01, P > 0.001 and P > 0.0001 compared to Col-0.

To further ascertain the role of COPT2 in the Au transport, elemental Au was measured in the tissues of mature Col-0 plants after four days of growth and compared with *copt2* mutant. The observation showed that a sharp and prominent decline of Au content occurred in the roots of *copt2* mutant compared to Col-0 (Fig. 3A). However, no significant difference was noticed in shoot tissues, suggesting that COPT2 may primarily be involved in Au uptake in the root rather than affecting root-to-shoot long distance transport (Fig. 3B). As anticipated, the Cu level was significantly decreased in the root of the *copt2* mutant compared to Col-0 under normal growing conditions, and was found similar to the Col-0 root after Au exposure (Fig. 3A). Interestingly, Cu content was higher in the shoot of the *copt2* mutant grown under control conditions with respect to Col-0 (Fig. 3B). Our results also indicated the same trend in Fe content within root and shoot grown under normal and treated conditions, except for Fe in the root of *copt2* mutant which was less relative to Col-0 under Au exposure (Fig. 3A). It has been shown that Cu and Fe homeostasis and their signaling are closely linked to each other in *Arabidopsis* ^17^. Further, the crosstalk of Cu, Fe, and P via the *COPT2*-mediated signaling event is an important realization based on past studies^17, 24^. Therefore, we have simultaneously measured the P level as well as other essential elements (Mn and Zn) in Col-0 and *copt2* mutant. The results showed that the accumulation of P and Mn content was found to be higher in root and shoot of *copt2* mutant compared to Col-0 under normal growing conditions while decreasing in root after Au exposure (Supplementary Figure 5). Yet, the level of Zn content was always found higher in the root and shoot of *copt2* mutant compared to Col-0 under normal and Au-treated conditions (Supplementary Figure 5). In this study, elemental profiling showed a significant reduction of Au content in the roots of *copt2* mutant with respect to Col-0 plants and explicitly confirmed that COPT2 mediated the uptake of Au in *Arabidopsis* similar to the Cu.

### Col-0 and *copt2* mutant growth similar in presence of Au

**Figure 4:**
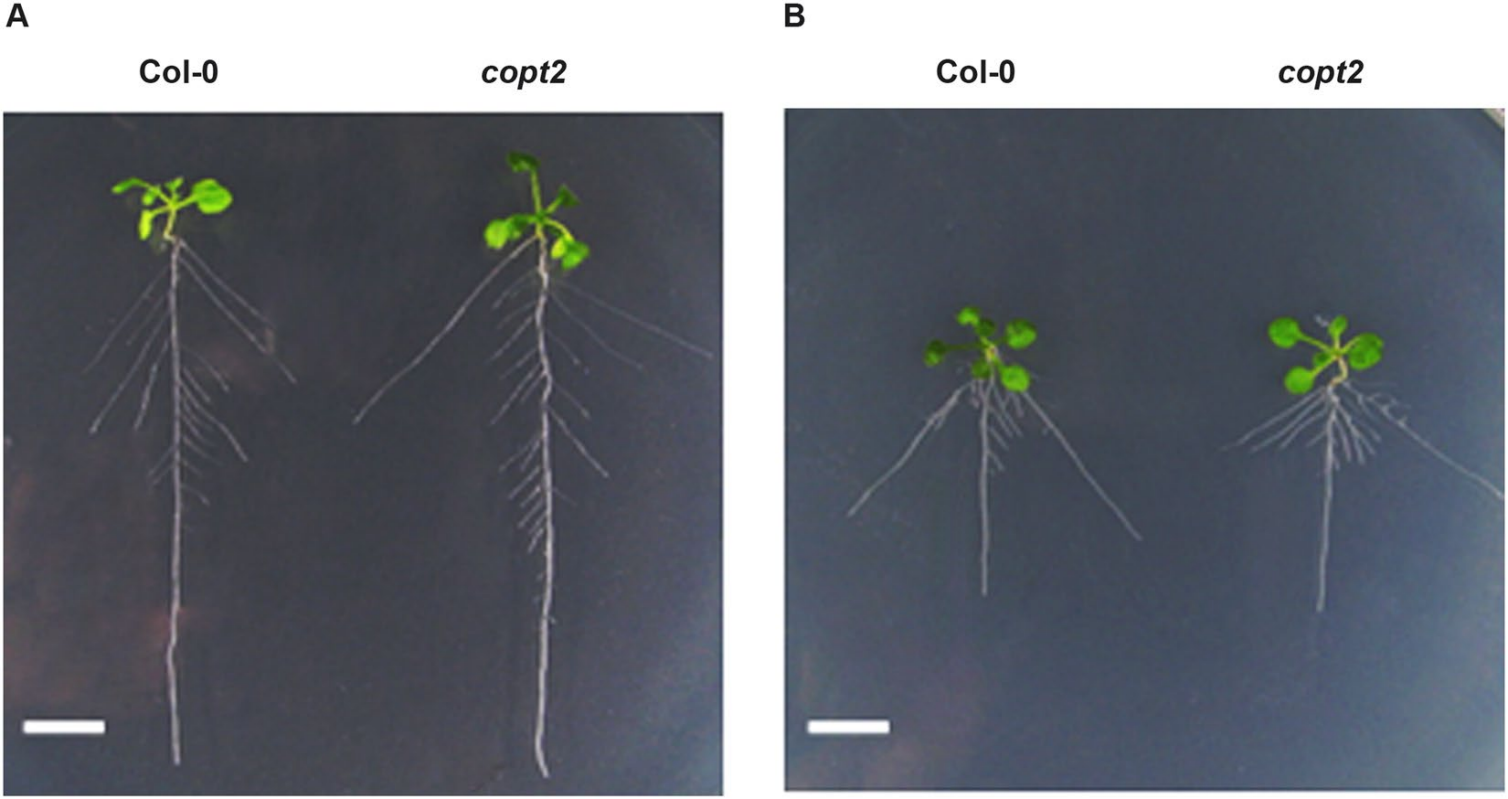
Effect of Au on *Arabidopsis* seedling growth. (**A**) Seedling growth and root architecture of Col-0 and *copt2* mutant grown under normal condition, and (**B**) exposure of Au (10 ppm). The white scale bar at the left bottom of panels represents 1 cm.

To know the effect of Au on the overall growth of *Arabidopsis* as well as any differences occurring by mutation of *COPT2*, wild type and *copt2* mutant seedlings grown for four days were further allowed to grow in the presence of 10 ppm Au in a hydroponic setup^9^. The seedlings growth was examined after one week of Au treatment. The observation indicated that Col-0 and the *copt2* mutant grew comparable to each other under normal conditions while the growth of the seedlings was significantly decreased in the presence of Au (Fig. 4A). However, there was no notable growth difference observed between Col-0 and *copt2* plants under Au treatment (Fig. 4B). The analysis of primary and secondary root length and the number of lateral root showed that the *copt2* mutant appears similar to Col-0 plants under Au exposure, suggesting that the mutation of *COPT2* was unable to create morphological changes in the root architecture of *Arabidopsis*. Nevertheless, the time-course exposure of Au using three-week-old mature plants revealed a distinct and visible change in the growth pattern of the *copt2* mutant compared to Col-0 plants after 2 and 4 days of Au treatment (Supplementary Figure 6). The dark green color of the *copt2* mutant was seen after 1 day of Au treatment in contrast to Col-0 where the distinct green coloration was observed after 2 days of Au exposure (Supplementary Figure 6). Moreover, significant less growth and biomass were visualized in the *copt2* mutant at 2 days and 4 days of Au treatment corresponding to Col-0 (Supplementary Figure 6A and B). Therefore, it has been deduced that the mutation of *COPT2* may alter the Au uptake of the *copt2* mutant and is linked to the direct role of COPT2 in the transporting of Au in *Arabidopsis*.

### High uptake of Au generates oxidative stress in Col-0

**Figure 5:**
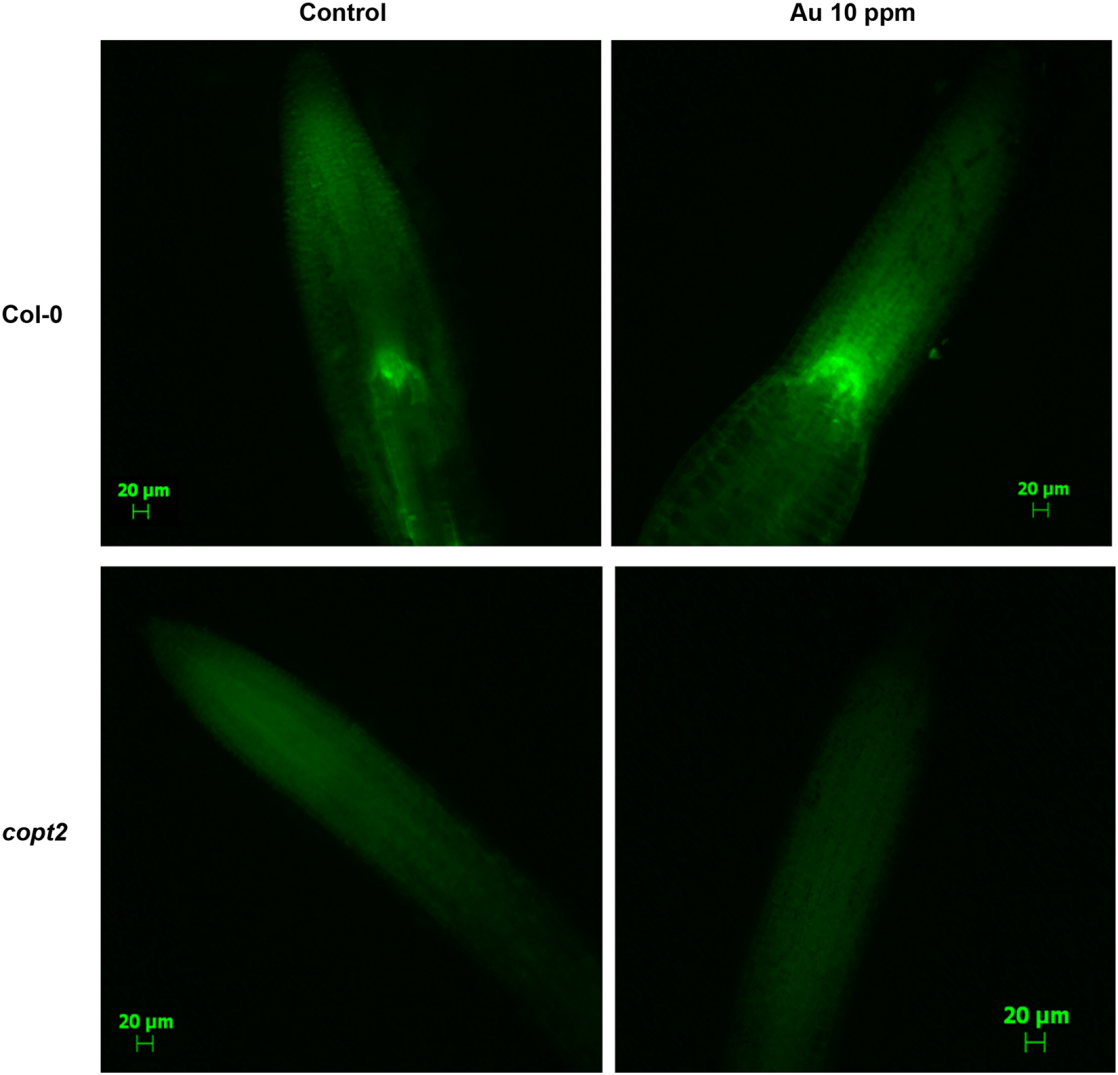
Au-induced ROS production at the root tip of *Arabidopsis*. The H2DCF staining of the root tip was performed under control and 48 h of Au treatment. The upper panels showing the Col-0 and the *copt2* mutant is in lower panels. The scale bars are equivalent to 20 µm.

The generation of reactive oxygen species (ROS) and oxidative stress is one of the common outcomes of abiotic or metal stress in plant. Our previous report also suggested that Au exposure consequently leads to the elevation of oxidative stress in the *Arabidopsis* ^9^. To know the effect of Au on cellular ROS production and whether impairment of the Au uptake process causes any alteration, a fluorometric-based assay was employed to measure the oxidative burst. Three-week-old mature plants were subjected to Au (10 ppm) stress for 48 h and fluorescence was monitored after staining with H2DCF in the root tip. The non-fluorescent compound H2DCF turned to a green fluorescent product when oxidized by peroxides. The amount of ROS generation under Au stress varied from plants grown in control conditions exemplified by fluorescence data and suggested that the high uptake of Au through functional COPT2 in Col-0 led to more oxidative burst in the root compared to the *copt2* mutant (Fig. 5). The results also indicate the lack of such fluorescence either in the control grown or under Au treatment in the *copt2* mutant (Fig. 5). The mutation of *COPT2* inhibited the movement of Au inside the root of the *copt2* mutant. Thus, a less ROS production occurred was seen in fluorescence imaging compared to Col-0. Finally, this data also indirectly supported the role of COPT2 in Au uptake in *Arabidopsis*.

## Discussion

The investigation of Au uptake in plant became an important research interest after the development of plant-based methods for biosynthesis of Au nanoparticles. The lack of knowledge about Au transport in plants and the probable role of COPT2 in this process identified in our previous reports encouraged us to conduct this study^9, 10^. The function of COPT2 has already been described for Cu uptake in yeast and *Arabidopsis*. It has also been shown that COPT2 plays a significant role in Fe and P deficiency responses^17^. The COPT2 displayed similar aerial expression patterns under Cu deficiency to that of COPT1 and complement *ctr1Δctr3Δ* mutants^16, 17^. In this study, our results clearly demonstrate that COPT2 can transport Au in the yeast mutant and *Arabidopsis*. Hence, this knowledge base could be used to enhance and refine plant-based nanoparticle synthesis methods in the future.

The gene expression analysis of all six members of the COPT family under Au exposure at different time intervals revealed the transcript accumulation of only *COPT2* in root after four days of treatment (Fig. 1). However, mRNA levels of *COPT2* and *COPT3* were also increased after 2 days of short Au exposure in shoot (Supplementary Figure 1). The COPT3 is a high-affinity Cu transporter and is well known to maintain the Cu homeostasis and plant fitness in *Arabidopsis.* ^25^. It is noteworthy that Au-induced specific expression of *COPT2* was observed among all members of the COPT family under long-term Au treatment. The result of time-course gene expression analysis is consistent with our previous report, which showed a significant upregulation of *COPT2* in roots under long-term (7 days) exposure of Au^9^. However, reports indicate that the *COPT2* mRNA level not only increased by Cu deficiency but also by Fe. In addition, it somehow linked with the P deficiency response as well^17^. The expression of COPT2 in yeast and spot assay performed on YPEG media resulted in typical Cu transport phenotypes as reported in earlier studies^13, 17^. Nonetheless, spot assay performed using SC-URA plates containing Au revealed a slight decrease in yeast cells expressing COPT2 compared to the vector-transformed control at higher dilutions (Supplementary Figure 2). This could be the result of an indirect effect of Au such as competition with Cu. To ensure that sensitivity came from more Au intake, we performed growth kinetics of yeast. The observation illustrated that COPT2-expressing cells were highly sensitive to the presence of Au in media and Au sensitivity at a higher dosage, which was comparable to Cu-induced growth inhibition (Fig. 2). We presumed that Au-induced growth inhibition in *COPT2*-expressing yeast cells was the consequence of more intake of Au. This assumption was confirmed by the measurement of Au in yeast cells after 4 h pulse treatment of Au, which revealed an accumulation several folds higher Au in *COPT2*-expressing yeast cells over vector control (Fig. 2). Interestingly, members of the CTR and COPT family have three transmembrane helices (TM1, TM2 and TM3) unlike most membrane-spanning transporters and are separated from each other by a cytoplasmic loop^14, 26^. It should be noted that members of the CTR family differ widely in size and degree of sequence similarity but have a well-defined domain structure^27^. Genetic evidence demonstrates that the occurrence of methionine rich motifs (MxxxM) at amino terminals of TM1 and TM2 plays an indispensable role in Cu acquisition and mediates proper metal coordination during the transfer process^14^. The biochemical and conformational studies showed that helices of CTR protein assemble in the form of symmetrical homotrimers that eventually result in a compact and novel channel architecture that permits highly specific Cu^+^ ions to move through the pores^26, 28–30^. Consistent with earlier studies, chloroaurate was used for Au treatment in this study. It has been shown that Au (°) and other oxidation forms of Au existed on root surfaces by the XANES spectra of alfalfa root samples^11^. Therefore, we propose that these forms of Au can be easily mobilized inside root cells through plasma membrane localized COPT2 protein.

The *copt2* mutant grew similar to Col-0 under normal as well as Au-exposed conditions, suggesting that the reduction of Au content in *copt2* mutant did not have an impact on seedling growth (Fig. 4). Nevertheless, notable differences such as less growth and more green coloration were noticed between the *copt2* mutant and Col-0 when the experiment was performed using mature (3 week-old) plants. The biomass of the *copt2* mutant was reduced under Au-supplied conditions after 2 and 4 days of Au exposure relative to Col-0 (Supplementary Figure 6), indicating less uptake of Au in mutant plants. Our previous reports showed that low concentrations of Au can induce plant growth, therefore, reduced growth of the *copt2* mutant might be due to less uptake of Au^9, 31^. The elemental analysis performed at the same conditions confirmed the manifold reduction of Au in the root of the *copt2* mutant with respect to Col-0, suggesting the direct role of COPT2 in Au transport (Fig. 3). The Cu level in the root of the *copt2* mutant was significantly decreased compared to Col-0 under normal conditions. However, Cu content in the shoot of the *copt2* mutant was higher compared to Col-0 (Fig. 3). The mutation of *COPT2* might affect Cu deficiency response signaling that subsequently altered the root-to-shoot distribution of Cu. It has already been reported that *COPT2* is not only responsible for Cu uptake but also showed crosstalk with essential elements such as Fe and P^17^. Besides these elements, Mn and Zn level were also found higher in the *copt2* mutant with respect to Col-0 and varied in both the root and shoot by Au exposure (Supplementary Figure 5).

The abiotic and exogenous metal stress often interfered with the electron transport system within mitochondria and chloroplast and, ultimately, become the source of elevated ROS production in plant. Our previous comparative proteome analysis of Au exposed in the root and shoot of *Arabidopsis* exhibited a change in the levels of crucial proteins involved in redox maintenance, indicating Au exposure resulted in high ROS production^9^. In that sense, it is noteworthy to measure internal ROS under Au exposure that was carried out using H2DCF in this study. The H2DCF staining of root tips clearly reflects Au-induced ROS production in Col-0 plants (Fig. 5). As anticipated, a less green fluorescence was seen in the *copt2* mutant compared to Col-0 due to less intake of Au ions, further strengthening our statement that COPT2 mediates the uptake of Au (Fig. 5). The monitoring of ROS generation in biological samples is a challenging task due to the instability of singlet oxygen and superoxide. The estimation of ROS mostly relies on the detection of chemiluminescence or fluorescence-based end product compounds, which have been formed by reaction with ROS^32^. The non-fluorescent substrates such as H2DCF produced a green fluorescent color after oxidation with ROS, especially H_2_O_2_, and is a widely used method for detecting intracellular ROS^32–34^. It has been suggested that H2DCF serves as an indicator of general oxidative stress rather than of only ROS production. However in any case, the lack of Au-induced oxidative burst in the *copt2* mutant was clear from H2DCF staining^32^. To reconcile our experimental data, we propose that owing to similar structure and chemistry, Au can easily pass through COPT2 into yeast and root cells of Col-0. The results of this study extend our knowledge of substrate specificity of COPT2 from the Cu to Au. Considering the Au accumulation property of certain plants, elucidation of Au transport mechanisms has a significant relevance as these plants could be used as an effective tool for the ‘phytomining’ of gold from Au-rich ores as well^35, 36^. This report has much significance for developing a strategy to enhance Au uptake for plant-based Au nanoparticles production or for the purpose of gold phytomining from Au ores rich sites.

## Methods

### Plant Materials and Growth Conditions

*Arabidopsis thaliana* ecotype Columbia-0 (Col-0) was used as a wild type. The *copt2* strain of *Arabidopsis* in which the *COPT2* gene mutated was also used in this study. The plants were grown in controlled conditions (120–140 mmolm^−2^ s^−1^ light intensity, 16/8 h light/dark cycle photoperiod and 230 C temperature). For the root architecture study, seedlings were grown in a hydroponic system as described in our earlier study^9^. Plants were grown in half-strength MS media (pH 5.7) containing 1% sucrose with and without CuSO_4_. For long-term, time-dependent growth assay, plants were grown in a hydroponic setup maintained in a tray for three weeks before treatments, as described by Conn *et al*.^37^ with little modifications such as half strength MS media (pH 5.7) was used for both germination and as the basal media^37^.

### Plasmid Construction and Yeast Experiments

The COPT2 open reading frame was amplified from the root cDNA library of Arabidopsis by PCR using gene specific primers: COPT2-BamH1-F5′-CATGGATCCATCATGGATCATGATCACATGCAT-3′, COPT2-EcoR1-R5′-AATGAATTCTGTTCAACAAACGCAGCCTGAA-3′ and cloned using the CloneJET PCR Cloning Kit (Thermo Fisher Scientific). Followed by sequence confirmation, the amplicon was further cloned in the yeast-expression vector (p426GDP) as described in an earlier study^13^. For Au uptake assay, *ctr1Δctr3Δ* the double mutant strain of *Saccharomyces cerevisiae* was transformed with the empty vector p426GPD or with the vector containing *COPT2* using a yeast transformation kit (Clontech). Yeast transformants were selected on synthetic complete media (SC-URA:0.67% yeast nitrogen base without amino acids, 0.2% dropout mixture without uracil, 2% glucose) and confirmed by colony PCR as described by Tiwari *et al*. (2014). For yeast spot assay, vector and *COPT2*-expressing yeast cells were grown up to 1.0 OD_600_ and following the serial dilution 10 µLof culture spotted on SC-URA 1.5% agar plates supplemented with 100 µM and 300 µM of KAuCl_4_, or without (served as control). The spot assay was also performed on YPEG media: 1% yeast extract, 2% bactopeptone, 2% ethanol, 3% glycerol and +Cu (100 µM CuSO_4_) and Au (50 and 200 µM KAuCl_4_) solidified with 1.5% agar. Plates were incubated for 3–4 days at 30 °C before being photographed. For growth inhibition assay, yeast cells were grown in 10 mL SC-URA liquid media up to 1.0 OD_600_ and a 10 µL primary culture was inoculated in 400 µL liquid media with and without Cu and Au and monitored the growth after every 1 h using Bioscreen C MBR (Bioscreen, Finland). The OD_600_ of culture devoid of any metals was considered as a reference during the relative percentage growth calculation.

### Root Architecture Analysis and Plant Growth Assessment

For short term growth assay, seeds were germinated on a half-strength MS medium including 1% sucrose for 4 days under sterile conditions in the hydroponic setup described in our previous studies^9, 31^. The seedlings were transferred in fresh half-strength MS media with and without 10 ppm KAuCl_4_ and allowed to grow for one more week before photographs were taken. For testing the Au effect on mature plants, three-week-old grown plants under hydroponic conditions were subjected to Au treatments for 4 days and photographed at indicated time intervals.

### RNA Extraction and Quantitative RT-PCR Analysis

The total RNA of samples was isolated from the root and shoot using a Spectrum™ Plant Total RNA Kit (Sigma-Aldrich, USA). Two micrograms of total RNA were subjected to DNase treatment (Promega) for removing residual genomic DNA and further used for cDNA synthesis by a SuperScript III First-Strand Synthesis kit (Invitrogen). The quantitative RT-PCR reaction was set up in a 10 μL total reaction mixture that contained 5 μL of 2× SYBR (ABI Biosystems, USA), 1 μL of the five-times diluted cDNA, 1 μL of forward and reverse primer (10 pM) and 2 μL nuclease-free water in a 96-well reaction plate in Applied Biosystems 7300 thermal cycler (Applied Biosystems, USA). The data was normalized using beta tubulin as the reference gene. Relative expression levels of the gene were calculated by the 2^−ΔΔCt^ method^38^. The primer sequences used for RT-PCR are described in Supplementary Table 1. The results were confirmed using three technical replicates of independent biological replicates.

### Elemental Determination

For elemental analysis in yeast, vector, and *COPT2*-expressing yeast, cells were grown initially in SC-URA media up to 1.5 OD_600_. Then, a pulse treatment of Au (100 µM) was provided for 4 h. After that, yeast cells were harvested by centrifugation and washed with nano-pure water at least 5 times by a series of centrifugation and re-dissolving cycles. Finally, yeast cells were dried at room temperature and 65 °C in a hot-air oven for 5 days, then digested in 5 mL of concentrated nitric acid at 85–90 °C for 3 days in a Pyrex tube. The samples were diluted 3 times and filtered with Whatman grade no. 1 filter paper. Individual metals were measured using an ICAP 6000 Series ICP Emission Spectrometer (Thermo Scientific).

For elemental analysis in Arabidopsis, three-week-old plants of Col-0 and *copt2* grown under hydroponic conditions were transferred in a tray having fresh half-strength MS (pH 5.7) medium supplemented with (10 ppm) and without Au. After 4 days of treatment, roots and shoots were carefully separated from each other. Following 4–5 washings of roots and shoots with nano-pure water, root tissues were kept in a desorption solution (2 mM CaSO_4_ and 10 mM ethylenediaminetetraacetic acid) for 10 min in order to remove surface and apoplastic Au contents^39^. The root tissue was further washed at least 5 times. The root and shoot samples were air dried at room temperature and later kept at 65 °C for 3 days in the oven. The acid digestion of each sample was carried out in 5 mL of concentrated HNO_3_ for 2 days at 80–85 °C. Samples were diluted 3 times with nano-pure water and filtered. Elemental content was measured using an ICAP 6000 Series ICP Emission Spectrometer (Thermo Scientific).

### ROS measurement

Arabidopsis plants were initially grown in the same hydroponic setup for 3 weeks. The media was replaced with fresh half-strength MS media (pH 5.7) containing Au (10 ppm). After 48 h of exposure, root samples were collected and washed with a 1× PBS buffer. The 2′,7′ dichlorofluorescein (H2DCF) (Sigma-Aldrich, USA) was dissolved in DMSO as 10 mM stock solution. Stock was diluted to make a final solution of H2DCF (10 µM in PBS buffer) in a dark eppendorf tube. The root samples were immersed in this solution for at least 10 min and then washed 3–4 times with 1× PBS buffer in the dark. The sample was mounted on a microscopic glass slide with 10% glycerol solution. The images were captured using an Epifluorescence microscope (Zeiss AxioPlan 2, Germany). To maintain the consistency of fluorescence among images, common default exposure time was set for all the captured images.

### Statistical Analysis

The yeast experiments, gene expression experiment, and root system analysis have been performed at least four times and the data of one set has been presented. Three biological replicates of each sample have been used in ICP-ES analysis. A student’s t-test was performed for comparison of mean and significance level determination.

## Electronic supplementary material

Supplementary Information
