# Supplementary material for: COPT2, a plasma membrane located copper transporter, is involved in the uptake of Au in Arabidopsis
Source: Sci Rep. 2017 Sep 12;7:11430. doi: 10.1038/s41598-017-11896-5 (PMC5595958; doi:10.1038/s41598-017-11896-5)

COPT2, a plasma membrane located copper transporter, is involved in the uptake of Au in *Arabidopsis*

Manish Tiwari[1#], Perumal Venkatachalam[2], Lola Penarrubia[3], Shivendra V. Sahi[1*]

[1]Department of Biology, Western Kentucky University,1906 College Heights, Bowling Green, 42101-1080 Kentucky, USA

[2]Department of Biotechnology, Periyar University, Salem 636 011, Tamil Nadu, India

[3]Departament de Bioquímicai Biologia Molecular Facultat de Biologia Universitat de València Ave. Doctor Moliner, 50 E-46100 Burjassot Valencia, Spain

[#]Present address: Department of Plant Systems Biology, VIB, Ghent University, Gent, 9000, Belgium

*****Corresponding Author; **Phone**: 270 745-6012 **Fax**: 270 745-6856

Supplementary Figure 1: Time dependent qRT-PCR analysis of *COPTs* in root and shoot of *Arabidopsis*. The bars and error bars show mean of three replicate and presented as ± SEM. Asterisks *, ** and *** placed on the top of the column denoting P>0.01, P>0.001 and P>0.0001.

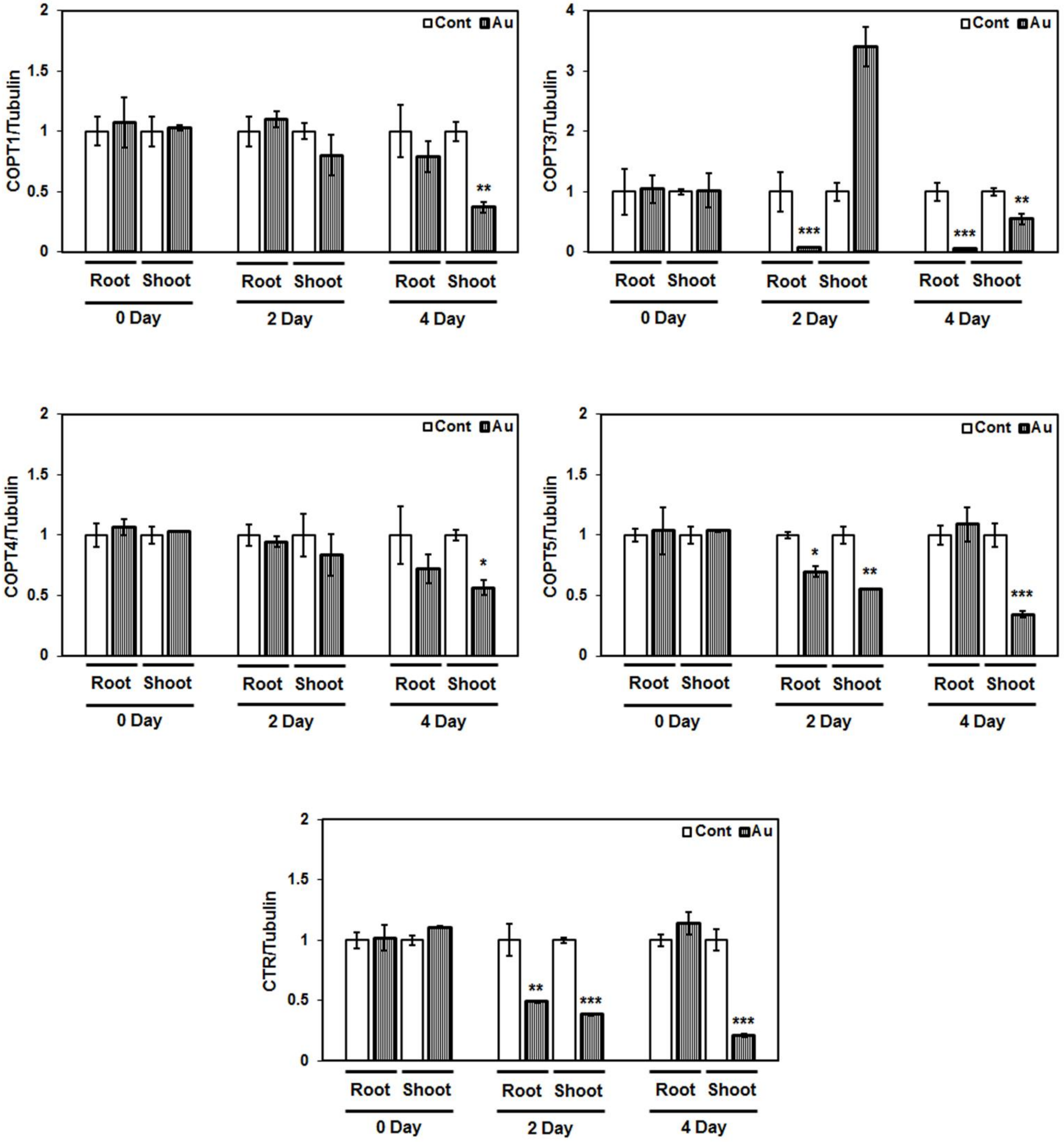

Supplementary Figure 2: Yeast spot assay carried out on YPEG media. Growth of *ctr1Δctr3Δ* on YPEG plate containing indicated amount of Cu (A) and Au (B). Each plate shows two independent yeast clones containing empty vector and COPT2 (left to right). The overnight grown culture used as preculture after maintaining the uniform growth (O.D. 1.0), and 10 µl aliquot of each serial dilutions plated down the plate (top to bottom).

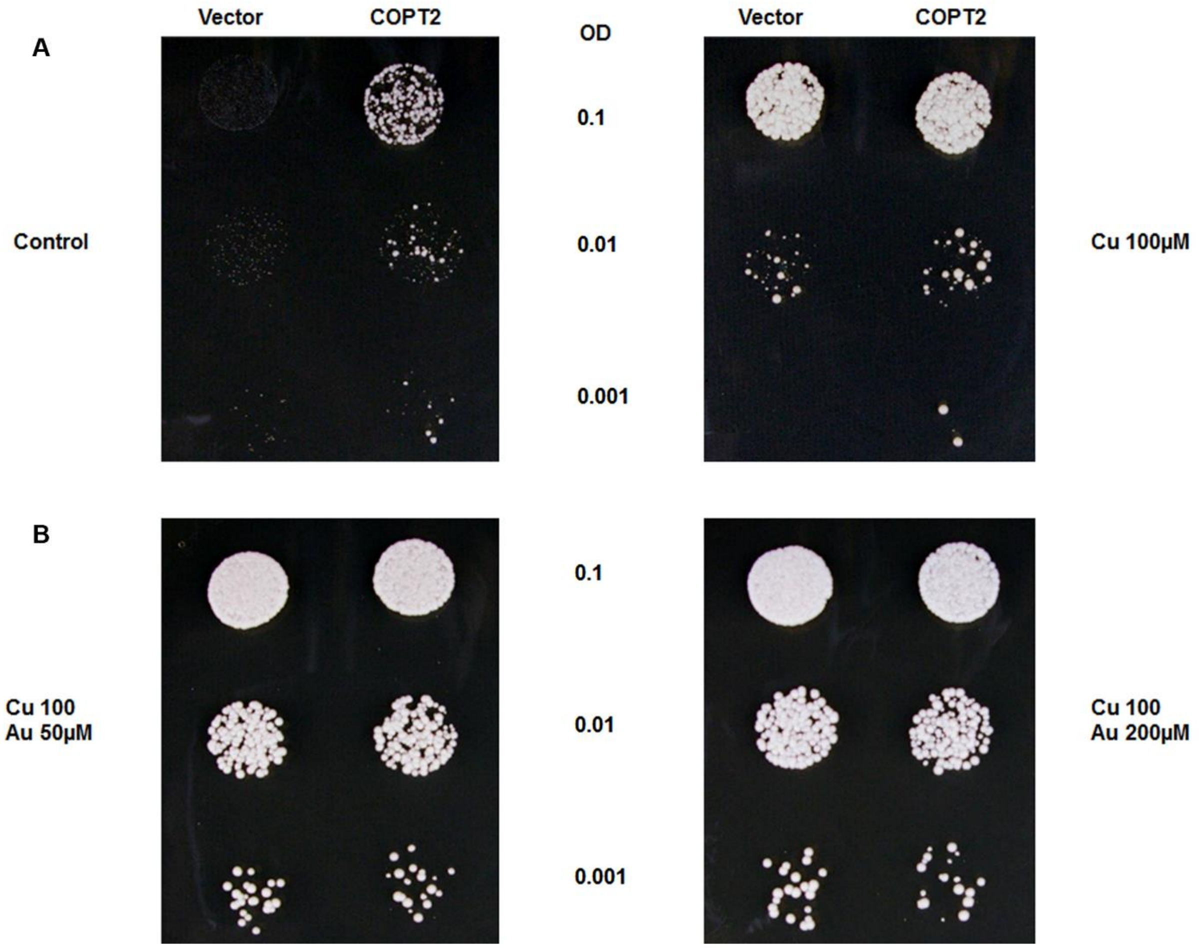

Supplementary Figure 3: Yeast spot assay showing sensitivity of yeast mutant towards Au. Growth of *ctr1Δctr3Δ*on SC-URA plate containing (left) 0, 100 and 300 μM Au (right). Each plate shows two independent yeast clones containing either an empty vector or COPT2 (left to right). The overnight grown culture used as preculture after maintaining the uniform growth (O.D. 1.0), and 10 μl aliquot of each serial dilutions plated down the plate (top to bottom).

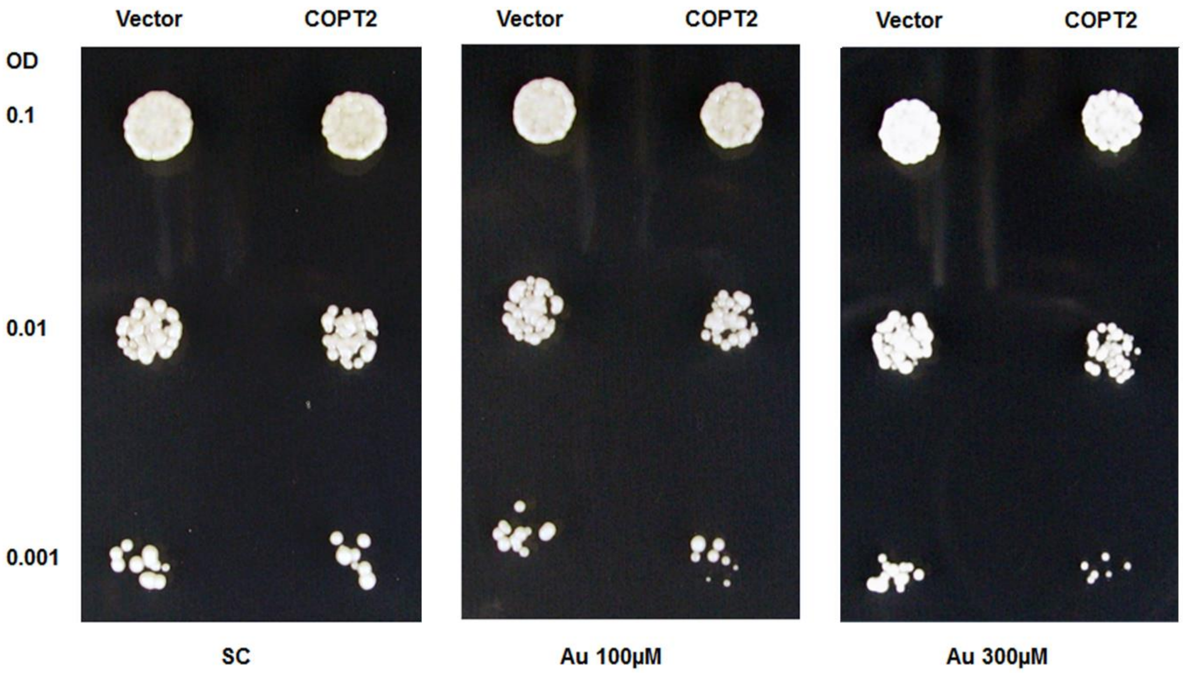

Supplementary Figure 4: Essential element measurement in yeast mutant. Overnight grown cultures (O.D. 1.5) administered with Au (100µM) and allowed to grow for 4 h. Cells were harvested and washed with nanopure water many times. Elemental profiling was performed using ICP-OES. Asterisks * and ** denote significant difference P>0.01 and P>0.001 compared to vector transformed yeast cells.

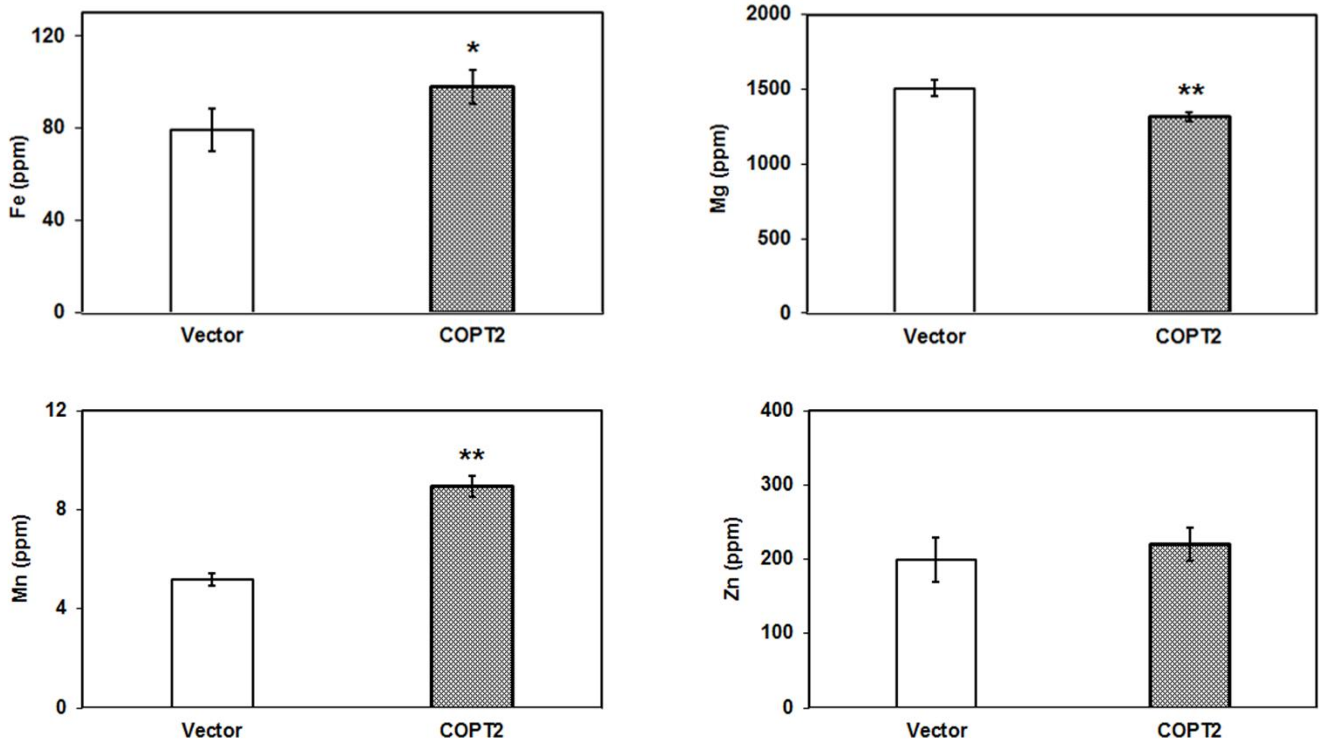

Supplementary Figure 5: ICP analysis of root and shoot of *Arabidopsis*. A) Elemental profiling carried out under normal and Au (10 ppm) treated conditions in root, and B) shoot. Data are mean of three independent biological replicates and presented as ± SEM. Asterisks *, ** and *** denote significant difference P>0.01, P>0.001 and P>0.0001 compared to Col-0.

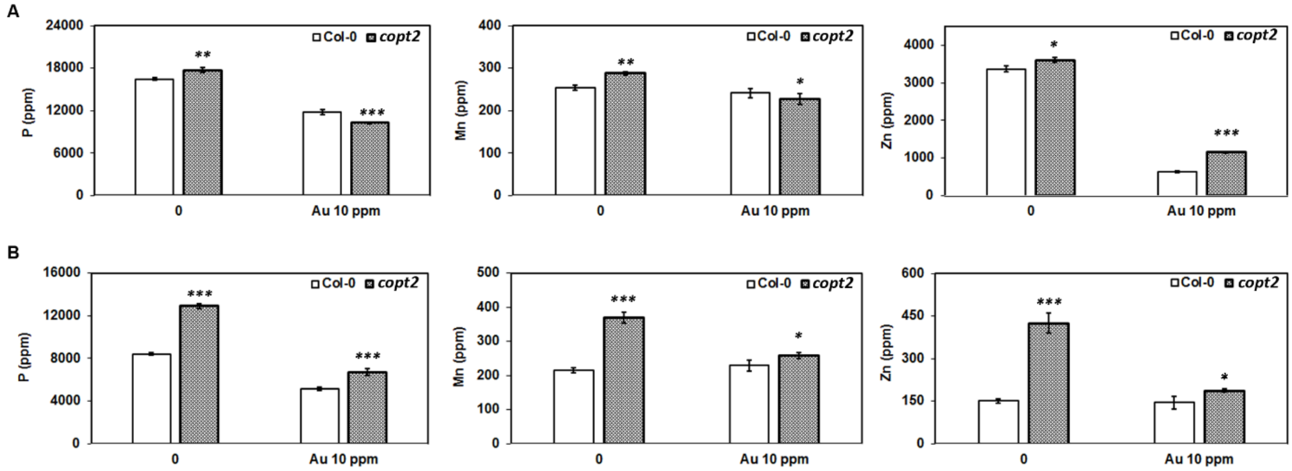

Supplementary Figure 6: Effect of Au on growth of mature plants. **A)** Growth of three week old Col-0 plants under control and Au (10 ppm) treatment at indicated time intervals, and **B)** Growth of three week old *copt2* mutant under control and Au (10 ppm) treatment at indicated time intervals.

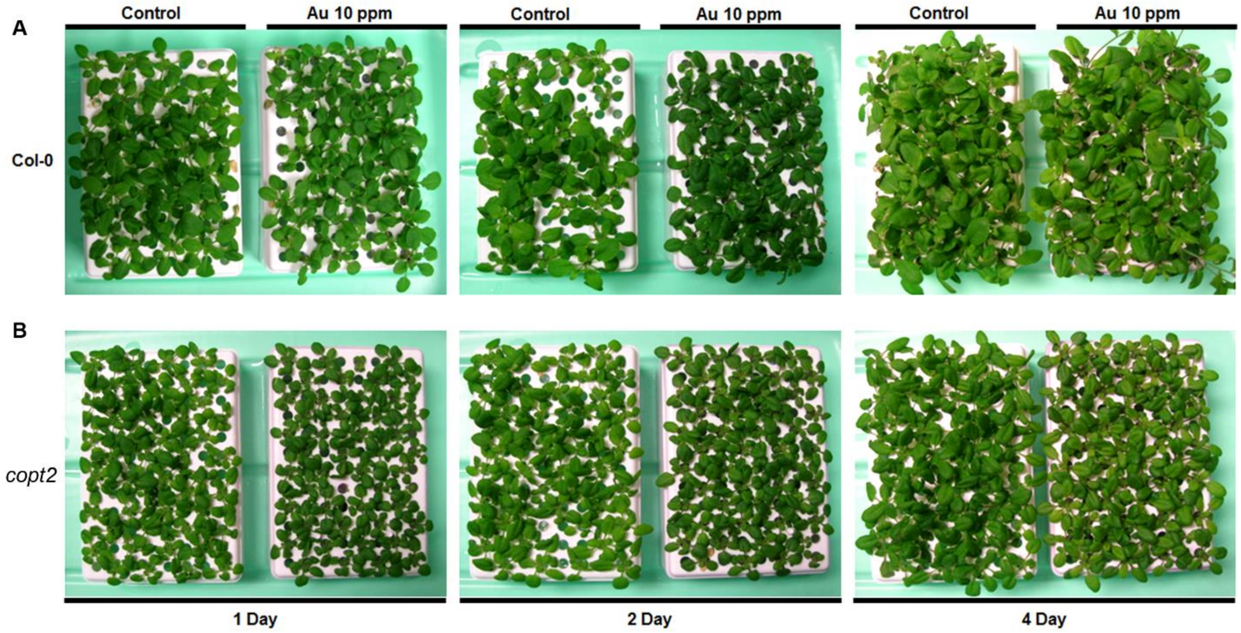

Supplement: Supplementary file 1 — Supplementary Information [file 41598_2017_11896_MOESM1_ESM.pdf]
